# A dataset of sea turtle occurrences around the Taiwan coast

**DOI:** 10.3897/BDJ.10.e90196

**Published:** 2022-11-03

**Authors:** Daphne Z. Hoh, Chia-Ling Fong, Huai Su, Pengyu Chen, Chia-Chen Tsai, Kelly W. H. Tseng, Melissa J. Y. Liu

**Affiliations:** 1 TurtleSpot Taiwan, Pingtung, Taiwan TurtleSpot Taiwan Pingtung Taiwan; 2 Taiwan Biodiversity Information Facility (TaiBIF), Biodiversity Research Centre, Academia Sinica, Taipei, Taiwan Taiwan Biodiversity Information Facility (TaiBIF), Biodiversity Research Centre, Academia Sinica Taipei Taiwan; 3 Biodiversity Research Centre, Academia Sinica, Taipei, Taiwan Biodiversity Research Centre, Academia Sinica Taipei Taiwan; 4 Biodiversity Program, Taiwan International Graduate Program, Academia Sinica and National Taiwan Normal University, Taipei, Taiwan Biodiversity Program, Taiwan International Graduate Program, Academia Sinica and National Taiwan Normal University Taipei Taiwan; 5 Independent Researcher, Taipei, Taiwan Independent Researcher Taipei Taiwan

**Keywords:** sighting data, citizen science, coastal waters, photo identification

## Abstract

**Background:**

We describe a dataset of sea turtle sightings around the coast of Taiwan and its islands (Hoh and Fong 2022). This data collection was initiated by TurtleSpot Taiwan, a citizen-science project that collects sea turtle sighting data. This dataset includes 3,515 sighting data dated from March 2010, except most of the data (n = 3,128; 89%) were collected between June 2017 to December 2021. Sightings were reported by citizen scientists to the Facebook Group of TurtleSpot Taiwan by providing occurrence information. We also requested photos and videos for species identification and to record any physical abnormality of the turtle, if observable. In addition to recording data often associated with an occurrence, TurtleSpot aims to identify each sea turtle up to the individual level using the Photo Identification (Photo ID) method. Hence, if photos of left facial scutes were available, the sighted individual can be identified and given a unique turtle ID. In total, 762 individuals were assigned a turtle ID, comprising 723 Greens (*Cheloniamydas*), 38 Hawksbills (*Eretmochelysimbricata*) and one Olive Ridley (*Lepidochelysolivacea*) turtle. This dataset is now publicly opened in Global Biodiversity Information Facility (GBIF) and available for download. It is hoped that the data may assist in future ecological studies and the development of conservation measures.

**New information:**

This dataset contains 3,515 occurrence records of sea turtles (Cheloniidae) and is currently the largest public dataset of sea turtle sighting records in Taiwan. Post-publication of this dataset to the GBIF platform demonstrated that the number of Green sea turtle *Cheloniamydas* records in Taiwan is one of the largest in the world (last accessed date: 15-10-2022). The data served as the foundation for understanding biogeography and sea turtle ecology in Taiwan's coastal waters.

## Introduction

People involved in citizen-science programmes have played a major role in contributing occurrence data of diverse organisms worldwide (Global Biodiversity Information Facility 2022, iNaturalist 2022). Following technological advancement, citizens can now report their sightings simply by using a mobile phone with an internet connection to biodiversity-associated citizen science platforms, such as iNaturalist and eBird. In addition, some citizen-science programmes are now incorporated with the use of social media for biodiversity data collection (Liberatore et al. 2018, Oliveira et al. 2021). One of the advantages of using social media platforms is the convenience and great opportunity for the recruitment and retention of volunteers and Facebook is the most widely used (Oliveira et al. 2021). For example, the Taiwan Roadkill Observation Network effectively use the Facebook Group for interactions within the community and collection of information, making it one of the most successful and active citizen-science projects in Taiwan.

Sea turtles are migratory Chelonian species that travel between nesting and foraging sites during their life cycle. In Taiwan, much is known about the nesting ecology (Cheng et al. 2009, King et al. 2013, Cheng et al. 2015, Cheng et al. 2018), owing to the easier accessibility of the nesting sites. Nonetheless, understanding of the foraging population and its ecology is still limited. Recognising the gap, TurtleSpot Taiwan - a community-led citizen-science initiative was founded to collect data with the majority focusing on the sighting of sea turtles underwater. This project started in 2017 and receives sighting reports provided by citizen scientists via the TurtleSpot Facebook Group. In addition to the sighting reports, TurtleSpot aims to develop a database of turtle profiles in Taiwan by identifying each individual turtle using the Photo ID method (Dunbar et al. 2021). We identified sea turtle individuals through their unique facial-scutes patterns and record any distinct characteristics of their physical appearances, such as carapace or limb injury, if available. To encourage continuous reports of the citizen scientists, we allow the sighting reporter to name the turtle if the individual is a new record in our database.

The purpose of preparing the current dataset was to publicly open the data for advancement, especially in the scientific and conservation communities. The data of TurtleSpot Taiwan have allowed a basic understanding in biogeography of foraging sea turtles in Taiwan and some ecological observations of sea turtles in the wild, such as witnessing the recovery of some injured turtles, types of behaviour, intra- and inter-species interactions and physical abnormalities.

## Sampling methods

### Step description

**Figure 1.**
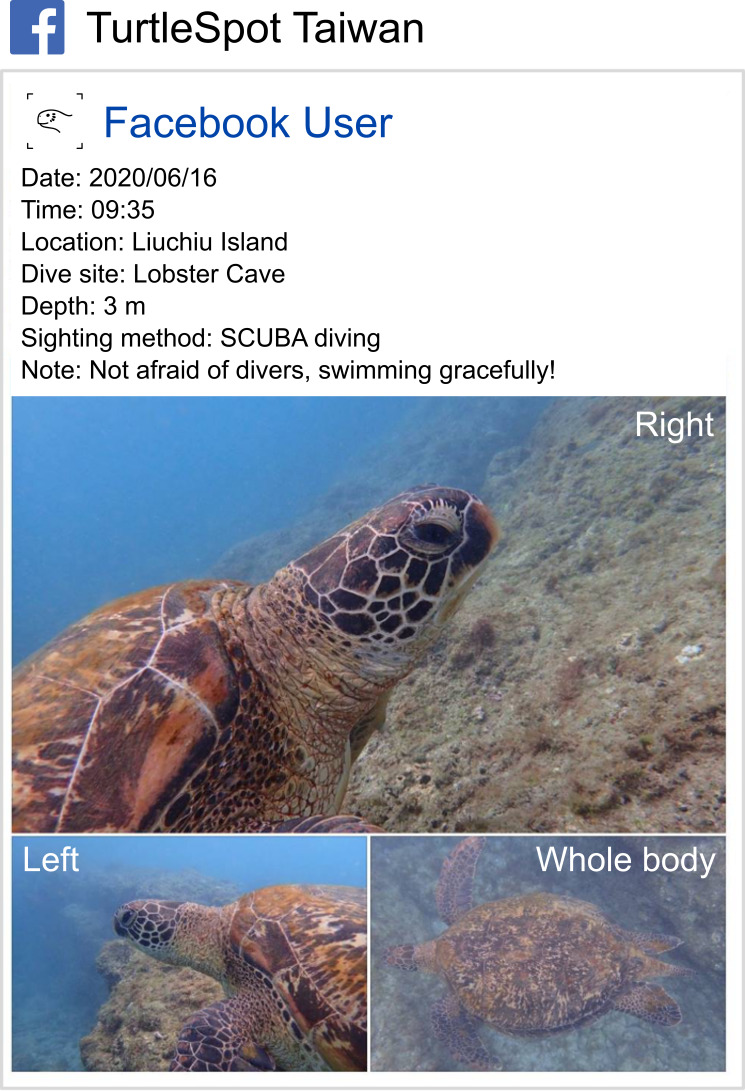
Format of reporting sea turtle sighting to TurtleSpot Taiwan. Facebook icon made by Freepik via Flaticon.

1. Data collection: Citizens who encountered sea turtles reported their sightings to us via our Facebook Group. Reporters post a regular post to the Group following our reporting format (Fig. 1) to contribute sighting information including sighting location, date, time, depth, observation method, photographs of the whole body and left- and right faces of the turtle individual.

2. Quality control of sighting report received: Each sighting reported to the Group was first checked by the group administration prior to approval. The group administration checked if the post followed the reporting format mentioned above and the sighting provider will be requested to provide any of the missing information unless unavailable. Once the submitted post passed the quality check, the post will be approved by the group administration to be visible in the Facebook Group.

3. Data transcription: Sighting information contained in the post/report was transcribed into Google Sheets as raw data.

4. Determine additional information from the sighting report: We recorded additional information about the occurrence through the sighting reporter’s notes of onsite observation and our identification through the provided photos and videos. Additional information included the biological characteristics of the sighted individual turtle (sex, life stage, behaviour, associated taxa) and physical abnormality of the turtle (e.g. fishing line entanglement, tumour and others).

5. Sea turtle individual identification: If clear photos of the left face of the sighted turtle were provided in the report, we use the Photo Identification (Photo ID) method to identify the turtle individual. Currently, we use two methods to perform Photo ID: (1) compare the facial scute pattern manually and (2) HotSpotter (Crall et al. 2013, Dunbar et al. 2021), open-source software for pattern recognition in wildlife research. Each sea turtle individual was assigned a unique turtle ID. The turtle ID was assigned as follows: Country code, site code, species code and sequence number. For example, in TW01G0082, “TW”, “01”, “G” and “0082” stands for Taiwan, island or county label, green turtle and unique number for the individual, respectively.

6. Open data preparation: The language used in most of the recorded data is Traditional Chinese. Nevertheless, valuable information including sighting location, method, common name and life stages which allowed future data use was translated into English. We converted the occurrence data into Darwin Core Archive standard in Google Sheets, an online spreadsheet tool, using the Darwin Core Archive Assitant Add-on (Salim and Saraiva 2020). Refer to the Data resources section for a detailed description of each column. We then validated the occurrence dataset using the Data Validator developed by GBIF (Global Biodiversity Information Facility 2017). Lastly, we uploaded, stored and published the dataset using The Integrated Publishing Toolkit (IPT) of GBIF installed under the Taiwan Biodiversity Information Facility. The data is then opened on the IPT and GBIF for the public to access.

## Geographic coverage

### Description

**Figure 2.**
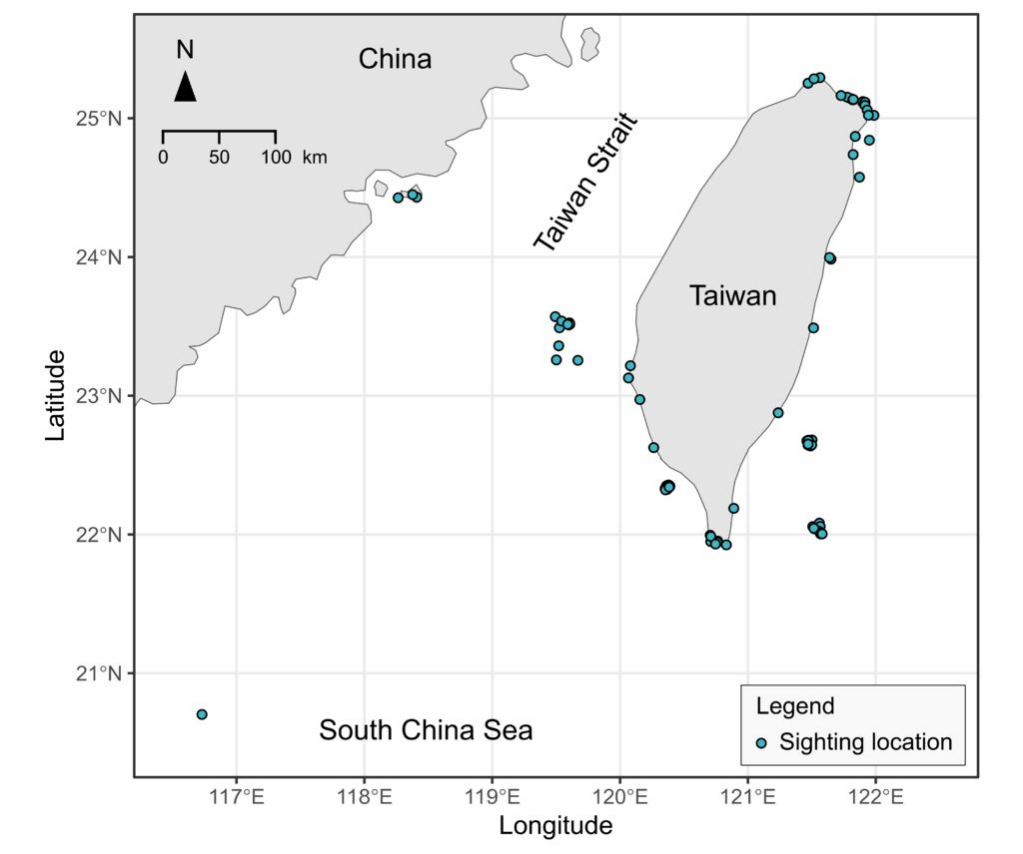
Location of sea turtle sightings in Taiwan. Data outside of Taiwan are not shown. Map was plotted using the R package 'rnaturalearth' (South 2017).

Most of the sighting data were from Taiwan and its islands (Fig. 2) and only a few (n = 35) were from other countries which include Indonesia, Philippines, Malaysia, Palau, the Mariana Islands, Japan, Maldives and United States.

## Taxonomic coverage

### Description

Four species of sea turtles were recorded in the dataset, including Green turtle (*Cheloniamydas*), Hawksbill (*Eretmochelysimbricata*), Olive Ridley (*Lepidochelysolivacea*) and Kemp's Ridley (*Lepidochelyskempii*). Most of the sea turtle sightings in the dataset were of Green and Hawksbill turtles (97.3% and 2.4%). Occurrences that failed to assign species (n = 11) were recorded as Cheloniidae.

### Taxa included

**Table:** 

Rank	Scientific Name	Common Name
kingdom	Animalia	Animal
phylum	Chordata	
subphylum	Vertebrata	
superclass	Reptilia	
order	Testudines	
suborder	Cryptodira	
superfamily	Chelonioidea	Sea turtle
family	Cheloniidae	
genus	Chelonia	
genus	Eretmochelys	
genus	Lepidochelys	
species	mydas	Green
species	imbricata	Hawksbill
species	olivacea	Olive Ridley
species	kempii	Kemp's Ridley

## Temporal coverage

**Data range:** 2010-3-23 – 2021-12-29.

### Notes

**Figure 3.**
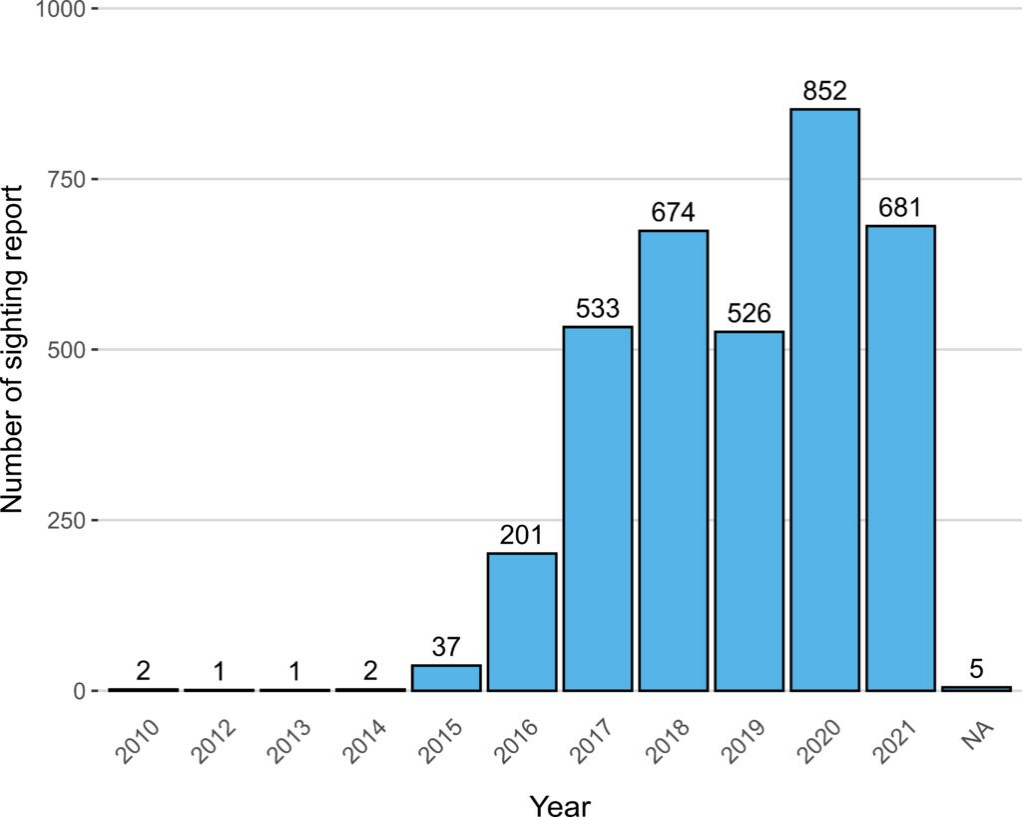
Number of sighting records across the year. 'NA' indicates no sighting year was given.

TurtleSpot was officially founded in June 2017. Hence, most sighting records range from June 2017 to December 2021, comprising about 89% (n = 3,128) of the dataset (Fig. 3). Occasionally, we receive sighting reports dated before June 2017, with the earliest dated 23 March 2010. Sighting reports dated before June 2017 were accepted and recorded if the sighting reporters could provide both photos/videos and occurrence information by following our reporting format as described in the Step description.

## Usage licence

### Usage licence

Other

### IP rights notes

The dataset in the current work is licensed under a Creative Commons Attribution (CC-BY) 4.0 Licence. Any image and video accessed through the URL from the dataset are licensed under the Creative Commons Attribution (CC-BY-NC) 4.0 Licence.

## Data resources

### Data package title

Sea turtle sightings in Taiwan

### Resource link

https://doi.org/10.15468/43z4mj

### Number of data sets

1

### Data set 1.

#### Data set name

Sea turtle sightings in Taiwan

#### Data format

Darwin Core Archive

#### Data format version

2021-07-15

#### Description

The dataset contains data of two major categories: data associated with the occurrence and data related to the biological characteristics of the sighted turtle individual. The former category consists of information during the sighting event such as date, time, location, geographical coordinates, observation method and species. The latter category characterised the observed turtle individual using our controlled vocabulary (see Suppl. material 1) during the sighting, including data such as living status, life stage, sex, physical abnormality and associated organism. The data allowed future research studies, such as biogeography, sea turtle foraging ecology that includes habitat use, sex ratio, abnormalities encountered and intra- and interspecies interaction. The data may also potentially guide any policy-making process through the assessment of species conservation status and diversity in the area of occurrences.

Some additional remarks on the dataset:

1. On average, 57 sighting reports were received monthly;

2. More than half (n = 2,235; 63.6%) of the data were provided by citizen scientists. The remaining data (n = 1,280; 36.4%) was records contributed by two of the co-authors as part of the citizen-science programme;

3. So far, only turtle sightings in Taiwan were given a turtle ID.

Data fields were standardised into 46 Darwin Core terms as listed in the following table. The column label and some of the relevant descriptions are written as listed in the List of Darwin Core terms (accessed June 2022; created by the TDWG Darwin Core Maintenance Group). A more specific description of the column used in the current dataset was also added if applicable.

The dataset is publicly opened in GBIF (see Resource link) and users can download the occurrence dataset in CSV format through the ‘Download’ section of the dataset page. The dataset can also be downloaded using GBIF API-based tools such as ‘rgbif’ and ‘pygbif’ for further analyses.

**Data set 1.** 

Column label	Column description
occurrenceID	An identifier for the Occurrence (as opposed to a particular digital record of the occurrence).
catalogNumber	An identifier unique for the record within the dataset.
rightsHolder	A person or organisation owning or managing rights over the resource.
recordedBy	Names of the sighting reporter/citizen scientist.
year	The four-digit year in which the Event occurred, according to the Common Era Calendar. Year of sighting.
month	The integer month in which the Event occurred. Month of sighting.
day	The integer day of the month on which the Event occurred. Day of sighting.
eventDate	Sighting date.
eventTime	The time or interval during which an Event occurred.
country	The name of the country in which the Location occurs.
countryCode	The standard code for the country in which the Location occurs.
higherGeography	A list of geographic names less specific than the information captured in the locality term.
locality	Name of the sighting location or dive site.
locationRemarks	More specific location compared to locality, usually the name of the dive site.
decimalLatitude	The geographic latitude (in decimal degrees, using the spatial reference system given in geodeticDatum) of the geographic centre of a Location. Positive values are north of the Equator, negative values are south of it. Legal values lie between -90 and 90, inclusive.
decimalLongitude	The geographic longitude (in decimal degrees, using the spatial reference system given in geodeticDatum) of the geographic centre of a Location. Positive values are east of the Greenwich Meridian, negative values are west of it. Legal values lie between -180 and 180, inclusive.
coordinateUncertaintyInMetres	The horizontal distance (in metres) from the given decimalLatitude and decimalLongitude describing the smallest circle containing the whole of the Location.
georeferenceRemarks	A note stating that our GPS coordinates were estimated from the dive site or sighting location.
geodeticDatum	The ellipsoid, geodetic datum or spatial reference system (SRS) upon which the geographic coordinates given in decimalLatitude and decimalLongitude are based.
verbatimDepth	The original description of the depth below the local surface. This is an estimation provided by the sighting reporter.
samplingProtocol	Sighting method of the Occurrence.
associatedReferences	An URL links to the Facebook post from the sighting reporter in our Facebook Group, which we define as a single Occurrence event. The link may be broken if the sighting reporter decided to delete the post.
basisOfRecord	The specific nature of the data record.
individualCount	Our purpose of preparing this occurrence dataset is to identify each turtle individual. Hence, if a sighting report contains more than one sea turtle, this occurrence record will be duplicated as a new row. Hence, the individual count of each data is only '1'.
kingdom	The full scientific name of the kingdom in which the taxon is classified.
taxonRank	The taxonomic rank of the most specific name in the scientificName.
vernacularName	A common or vernacular name.
scientificName	The full scientific name.
taxonID	An identifier for the set of taxon information (data associated with the Taxon class). We use the URL of species in GBIF Backbone Taxonomy checklist.
behaviour	The behaviour shown by the subject at the time the Occurrence was recorded.
occurrenceRemarks	Condition of the turtle during the sighting (e.g. alive, dead, stranded).
dynamicProperties	Any physical abnormality that was observed (e.g. injury, tumour, debris entanglement).
associatedTaxa	A simple description of association and vernacular name of taxa in which this Occurrence is to each of them.
lifeStage	The age class or life stage of the organism at the time the Occurrence was recorded. Estimated via physical appearence of the sighted turtle.
sex	The sex of the biological individual represented in the Occurrence. Determination of sex is only applicable to adult sea turtles through the size of their tail. Sex determination is successful only when photos/videos of the tail are available.
organismName	A textual name or label assigned to an Organism instance. Mostly named by the citizen scientists.
licence	A legal document giving official permission to do something with the resource. The licence in this column is applied to the text data of this dataset only.
identificationID	Turtle ID. Every identifiable turtle individual has a unique ID.
associatedMedia	An URL links to the website of TurtleSpot Turtle Photo ID database, which show media and information about this particular individual.
identifiedBy	Names of people who identified the turtle individual via Photo-ID method.
informationWithheld	Additional information that exists, but that has not been shared in the given record.
occurrenceStatus	A statement about the presence or absence of a Taxon at a Location. All value is 'present' in the current dataset.
eventRemarks	Notes about the incomplete sighting eventDate.
continent	The name of the continent in which the Location occurs.
county	The full, unabbreviated name of the next smaller administrative region than stateProvince (county, shire, department etc.) in which the Location occurs.
island	The name of the island on or near which the Location occurs.

## Supplementary Material

73D1893C-E80E-5244-AD68-86DFAF9CD2C3 10.3897/BDJ.10.e90196.suppl1 Supplementary material 1 Controlled vocabulary describing additional information about the occurrence Data type biological File: oo_757953.csv https://binary.pensoft.net/file/757953 Chia-Ling Fong & Daphne Z Hoh
